# Regional Variability of Extreme Heat and Cold Risk Among Dual-Eligible Individuals

**DOI:** 10.1001/jamahealthforum.2024.5134

**Published:** 2025-01-24

**Authors:** Eun-Hye Yoo, Clint Sergi, Angela Senders, Hyunjee Kim

**Affiliations:** 1 Department of Geography, University at Buffalo, Buffalo, New York; 2 Center for Health Systems Effectiveness, Oregon Health & Science University, Portland

## Abstract

This cross-sectional study identifies associations between emergency department visits and extreme temperatures across US geographic regions.

## Introduction

The health burden of extreme temperature is considerable and is expected to increase. While previous studies have highlighted devastating health outcomes of extreme temperatures,^1,2^ empirical evidence on how these vulnerabilities vary across geographic regions remains limited. To address this gap, we examined the associations between extreme temperatures, both hot and cold, and all-cause emergency department (ED) visits across different climate zones in the US. We focused on Medicare-Medicaid enrollees (dual-eligible individuals) aged 65 years or older, a population particularly susceptible to extreme temperatures due to a high prevalence of chronic health conditions and on average lower socioeconomic status.^3^

## Methods

Our cross-sectional study included 24 690 178 all-cause ED visits from 2016 through 2020 among dual-eligible individuals across the 9 contiguous US climate regions identified by the National Centers for Environmental Information (Northeast, Northern Rockies and Plains, Northwest, Ohio Valley, South, Southeast, Southwest, Upper Midwest, and West).^4^ We applied a time-stratified case-crossover study design with a distributed lag nonlinear modeling framework to estimate associations between daily mean temperature and ED visits for each region.^5,6^ We calculated the relative risks (RRs) of all-cause ED visits associated with extreme cold (2.5th temperature percentile) and heat (97.5th temperature percentile), accounting for potential delayed effects over extended lag periods (eMethods in Supplement 1). R software (version 4.3.3; R Core Team) was used for analysis. Results were considered statistically significant at *P* < .05, and tests were 2 sided. The Oregon Health & Science University Institutional Review Board approved this study, and we followed the STROBE reporting guideline. Informed consent was unnecessary because data were deidentified.

## Results

Exposures to extreme hot and cold temperatures were associated with consistently higher rates of all-cause ED visits, but with notable regional variability. The region temperatures are reported in the Table. Heat exposure was significantly associated with increased number of ED visits in all 9 regions, but the RRs were highest in the Upper Midwest (RR, 1.22; 95% CI, 1.19-1.25), Northern Rockies and Plains (RR, 1.21; 95% CI, 1.14-1.28), and Northwest (RR, 1.20; 95% CI, 1.15-1.25) (Figure, A). Conversely, the RRs for extreme cold and ED visits were highest in the South (RR, 1.21; 95% CI, 1.18-1.24), Southeast (RR, 1.14; 95% CI, 1.12-1.16), and Southwest (1.12; 95% CI, 1.03-1.22) (Figure, B).

**Table.** Total Number of All-Cause ED Visits and Daily Mean and Median Temperature by US Climate Region (2016-2020)

Region	All-cause ED visits, No. (%)	Daily temperature, °C	
		Mean (range)	Median (2.5th-97.5th percentile)
Northeast	5 536 369 (22.42)	10.1 (−25.7 to 32.1)	10.4 (−9.7 to 26.2)
Northern Rockies and Plains	226 813 (0.92)	7.6 (−33.6 to 31.9)	7.8 (−16.6 to 25.6)
Northwest	831 215 (3.37)	9.3 (−21.6 to 30.8)	9.2 (−7.0 to 23.9)
Ohio Valley	3 793 488 (15.37)	13.0 (−28.8 to 32.4)	13.7 (−6.8 to 27.1)
South	3 622 495 (14.67)	17.5 (−19.8 to 36.1)	18.8 (−1.4 to 30.2)
Southeast	5 101 795 (20.66)	17.5 (−16.5 to 32.2)	18.9 (0.5-28.8)
Southwest	897 889 (3.64)	10.5 (−23.1 to 39.1)	10.5 (−8.2 to 27.6)
Upper Midwest	1 482 518 (6.00)	7.7 (−34.9 to 30.7)	8.1 (−16.3 to 25.2)
West	3 197 596 (12.95)	14.5 (−16.2 to 39.3)	14.7 (−1.3 to 28.5)

Abbreviation: ED, emergency department.

**Figure.**
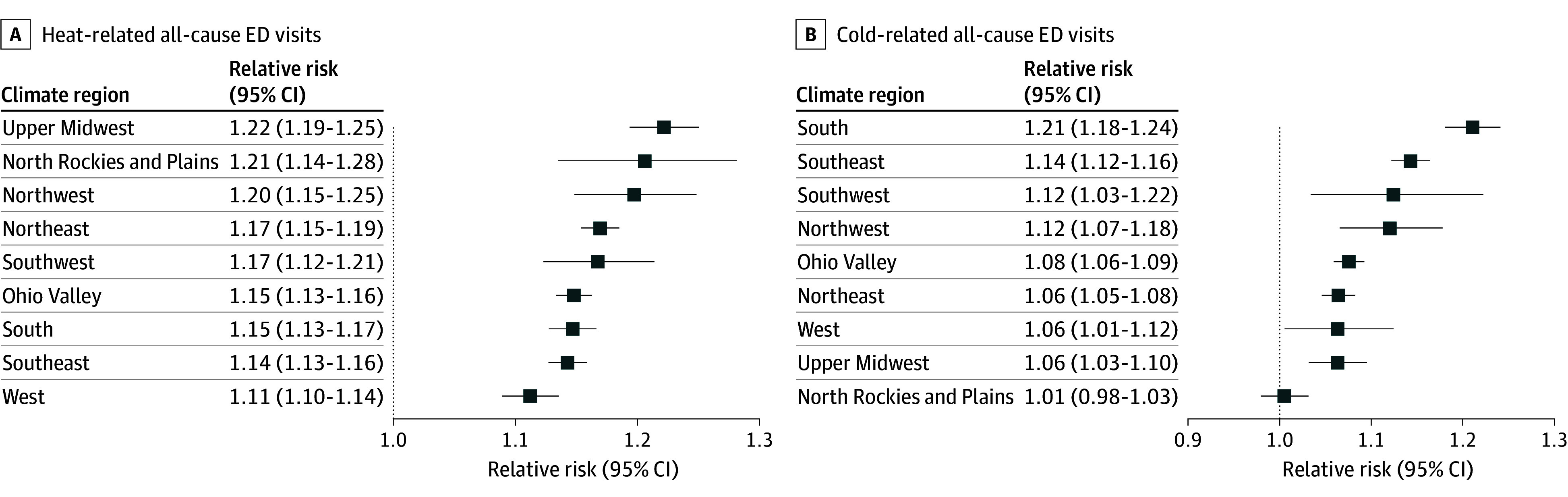
Region-Specific Relative Risks of All-Cause Emergency Department (ED) Visits Associated With Extreme Heat and Cold in the US, 2016-2020 Region-specific relative risks and 95% CIs of all-cause ED visits cumulated over a 3-day lag period associated with hot temperature (97.5th percentile) (A) and a 21-day lag period associated with cold temperature (2.5th percentile) (B) in 9 regions from 2016 through 2020.

## Discussion

Our study found that both extreme heat and cold were associated with increased number of ED visits among dual-eligible individuals aged 65 years or older. However, regions at higher latitude, such as the Upper Midwest, the Northern Rockies and Plains, and the Northwest, were most at risk from extreme heat while southern regions, including the South, Southeast, and Southwest, were more susceptible to extreme cold. The fact that higher-latitude regions were more susceptible to extreme heat while lower-latitude regions faced greater risk from extreme cold suggests that susceptibility to temperature extremes may be shaped more by unpredictable weather patterns and insufficient preparedness than by geographic location alone. The study had several limitations, including the potential misclassification of temperature exposure due to the inability to account for indoor temperature or individuals’ time-activity patterns. Additionally, unmeasured meteorologic factors may have introduced residual confounding effects.

Our study underscores an urgent need to advance and update region-specific climate adaptation and resilience plans, building on efforts already under way by organizations such as the National Integrated Heat Health Information System. Enhancing public health campaigns to raise awareness among vulnerable populations about the risks of extreme temperatures and effective preventive measures is essential. Additionally, fostering community resilience programs that strengthen local preparedness can play a pivotal role in mitigating the health outcomes of extreme weather events and safeguarding at-risk populations.

## Notes

Supplement 1.
eMethods

eReferences

Supplement 2.
Data Sharing Statement
