# Supplementary material for: Regional Variability of Extreme Heat and Cold Risk Among Dual-Eligible Individuals
Source: JAMA Health Forum. 2025 Jan 24;6(1):e245134. doi: 10.1001/jamahealthforum.2024.5134 (PMC11762227; doi:10.1001/jamahealthforum.2024.5134)
Supplement: Supplement 1. — eMethods eReferences [file jamahealthforum-e245134-s001.pdf]

Supplemental Online Content

Yoo E-H, Sergi C, Senders A, Kim H. Regional variability of extreme heat and cold risk among dual-eligible individuals. *JAMA Health Forum*. 2025;6(1):e245134.
doi:10.1001/jamahealthforum.2024.5134

eMethods. A Time-Stratified Case-Crossover Analysis Using Distributed Lag Non-Linear Models by US Climate Regions

eReferences

This supplemental material has been provided by the authors to give readers additional information about their work.

© 2025 Yoo E-H et al. *JAMA Health Forum*.

A Time-Stratified Case-Crossover Analysis Using Distributed Lag Non-Linear Models by US Climate Regions

In a time-stratified case-crossover design,[1] individuals serve as their own control, and inferences are based on the comparison of exposure (i.e., daily mean temperature) of the case period to the exposure of control periods that are closely matched to the case period. In the present study, we chose control periods from the same calendar month as the case period (i.e., the time when an emergency room visit was made), which ensures that both the case and control periods fall within the same seasonal cycle. Therefore, long-term temporal trends, seasonality, day of week, and potential time-invariant confounders, such as the individual's age, sex, socioeconomic conditions (i.e., housing conditions and access to air conditioning or heating), and other fixed participant characteristics (e.g., chronic health conditions) are efficiently controlled. The daily mean temperature for each case and control was determined from the daily surface weather data (Daymet[2]) by averaging 1km x 1km daily mean temperature values from all grid cells that are within or that intersect within a county boundary.

We also used a distributed lag nonlinear modeling (DLNM) framework to capture non-linear exposure-response relationships while accounting for delayed effects over extended lag periods. Model details regarding DLNM is described in Gasparrini [3]. We modeled exposure-response associations using a cross-basis function with two knots placed at equal intervals on the log scale of lags. We examined the impact of exposure to heat and cold separately, considering both short-term and delayed effects (i.e., up to several days or weeks post-exposure). The consideration of delayed effects is crucial because health effects of temperature extremes may not manifest immediately and could occur several days or weeks after the exposure, particularly for cold temperatures. To determine the robustness of our results, we conducted a set of sensitivity analyses across different lag structures and concluded that the best model fit was obtained at cumulative lag period of 3 days for heat effects and 21 days for cold effects.

The relative risks and 95% confidence intervals were estimated using conditional logistic regression by 9 climate regions, comparing all-cause ED visits associated with extreme temperature, which was defined as temperature lower than the 2.5th region-specific percentile ('extreme cold') and higher than the 97.5th region-specific percentile ('extreme heat'), with region-specific minimum all-cause ED visits temperature. The sensitivity of the results was assessed using other definitions of extreme temperature (i.e., cutoffs at the 1st, 5th, 95th, 99th percentiles), and we found that our results for the top 3 regions being most vulnerable for cold and heat effects were consistent regardless of the cutoff values.

© 2025 Yoo E-H et al. *JAMA Health Forum*.

The 9 climate regions were defined by National Centers of Environmental Information, which consists of the Northwest (WA, OR, ID), the Northern Rockies and Plains (MT, WY, ND, SD, NE), the Upper Midwest (MN, WI, MI IA), the Northeast (NY, PA, MD, DE, NJ, CT, MA, RI, VT, NH, ME), the West (CA, NV), the Southwest (UT, CO, AZ, NM), the South (KS, OK, AR, MS, LA, TX), the Southeast (VA, NC, SC, GA, AL, FL), and the Ohio Valley (MO, IL, IN, KY, TN, OH, WV).

Reference

1. Janes H, Sheppard L, Lumley T. Case–crossover analyses of air pollution exposure data: referent selection strategies and their implications for bias. *Epidemiology*. 2005;16(6):717-726.
2. Thornton MM, Shrestha R, Wei Y, Thornton PE, Kao SC. Daymet: Daily Surface Weather Data on a 1-km Grid for North America, Version 4 R1. ORNL Distributed Active Archive Center; 2022.
3. Gasparrini A. Modeling exposure–lag–response associations with distributed lag non-linear models. *Statistics in medicine*. 2014;33(5):881-899.

© 2025 Yoo E-H et al. *JAMA Health Forum*.
