# Supplementary material for: Regional Variability of Extreme Heat and Cold Risk Among Dual-Eligible Individuals
Source: JAMA Health Forum. 2025 Jan 24;6(1):e245134. doi: 10.1001/jamahealthforum.2024.5134 (PMC11762227; doi:10.1001/jamahealthforum.2024.5134)
Supplement: Supplement 2. — Data Sharing Statement [file jamahealthforum-e245134-s002.pdf]

Data Sharing Statement

Yoo. Regional Variability of Extreme Heat and Cold Risk Among Dual-Eligible Individuals. *JAMA Health Forum*. Published January 24, 2025. doi:10.1001/jamahealthforum.2024.5134

Data
Data available: No
